# PMconv: How to Compare Proteomes and Metabolomes?

**DOI:** 10.3390/ijms27115086

**Published:** 2026-06-04

**Authors:** Anna Kozlova, Anna Kliuchnikova, Arina Gordeeva, Andrey Lisitsa, Elena Ponomarenko, Ekaterina Ilgisonis

**Affiliations:** Institute of Biomedical Chemistry, 119121 Moscow, Russia; ministreliya13113@gmail.com (A.K.); a.kliuchnikova@gmail.com (A.K.); arina.atom@gmail.com (A.G.);

**Keywords:** proteometabolomics, mass-spectrometry, data integration, multi-omics

## Abstract

Integrating proteomic and metabolomic data remains challenging due to the many-to-many relationships between metabolites and proteins and the spatial constraints of cellular compartmentalization. To address this, we developed PMconv, a web-based application for bidirectional, knowledge-based mapping of proteomic and metabolomic datasets. Leveraging curated associations from the Human Metabolome Database (HMDB) and protein interaction data from STRING, PMconv infers potential biochemical connections between experimentally detected molecules and pathway-annotated partners. The tool supports interactive network visualization and exports compartment annotations from the Human Protein Atlas to facilitate spatial contextualization of inferred interactions. PMconv is designed as an exploratory resource for hypothesis generation and feature engineering in multi-omics research, with the explicit understanding that knowledge-derived associations require experimental validation for compartment-specific interpretation.

## 1. Introduction

Mapping molecular profiling results onto biological pathways reveals systemic changes in molecular processes [1]. The completeness of the results depends on the quality of data integration and information about various levels of implementation of genetic information (genomic, transcriptomic, translatomic, proteomic and metabolomic), as well as the degree of influence of regulatory processes, the presence of multiple protein forms encoded by one gene (proteoforms), and post-translational modifications and epigenetic modifications of DNA/RNA.

Transcriptomic, translatomic, and proteomic databases (e.g., Ensembl, TranslatomeDB, UniProt) are characterized by a gene-centric [2] data model, in which qualitative and quantitative measurements are mapped to protein-coding genes on chromosomes. The gene-centric approach makes it possible to integrate information about genes and their products obtained from high-throughput experiments at the transcriptome, translatome, and proteome levels. Difficulties arise when comparing gene-centric and non-gene-centric data: for example, a list of metabolites found in a sample. Each of the metabolites cannot be uniquely associated with only one gene due to the peculiarities of biochemical processes, since one metabolite can be produced by several enzymes.

Metabolomics databases, such as the Human Metabolome Database (HMDB) [3] and KEGG [4], use a metabolite-centric data model, in which a metabolite is associated with a list of proteins (enzymes, receptors and transporters) that can be uniquely associated with the coding gene within the framework of a gene-centric model. A protein is a connecting object in a chain of gene–protein–metabolite relationships.

One of the possible options for establishing relationships between objects in this chain is projection onto metabolic pathways. A number of analytical resources, for example IMPaLA [5], Metscape [6], paintOmics [7], KEGG [4] and MetaboAnalyst [8], are based on the conversion of gene, protein, transcript, and metabolite identifiers into a metabolic pathway identifier (for example, KEGG ID and/or Reactome ID). A list of both identified proteins and metabolites can be associated with a set of metabolic pathways.

Over the past ten years, a few works have been published that consider the proteome and metabolome as one entity—the proteometabolome [9,10,11,12,13,14]. While several tools exist for proteometabolomic analysis, there remains a need for user-friendly platforms that facilitate bidirectional protein–metabolite mapping based on pathway associations, as well as tools for reconstructing protein–metabolite interactions. Existing systems are limited to a narrow range, mainly to proteins and metabolites associated with drug action. With such a restriction, a significant part of the interactions between proteins and metabolites remains beyond the access of the researcher.

An example of a proteometabolomic information resource is the MetalinksDB [15]. This is a database that integrates information on relationships between proteins and metabolites from HMDB, UniProt and several other resources. The visualization system allows users to customize filters, displaying relationships specific to a tissue or pathological process. MetalinksDB provides static database queries without integrated network visualization of experimentally obtained proteomic and metabolomic data.

We have proposed a software solution for interpretation and visualization in the form of a network of interactions of the results of proteomic and metabolomic profiling.

We propose an enrichment method using identifier conversion by the functionality of the HMDB resource. Our program allows converting metabolites to proteins and vice versa, through belonging to the same metabolic pathway. Conversions of this kind can be carried out iteratively and repeatedly, each time increasing the number of relationships and associated objects.

Reconstruction of protein–metabolite networks based on the results of experimental profiling of blood plasma can help to identify the fraction of proteins and metabolites associated with pathological processes of cells and tissues, as well as the major proteomic fraction of blood. Associations established in this way between proteins and metabolites are new, non-obvious characteristics of molecular processes, including pathological ones. Current proteometabolomic analysis faces a fundamental challenge: while experimental datasets capture tissue-specific molecular snapshots, biological interpretation requires integrating these observations with the broader landscape of known biochemical relationships. Existing pathway analysis tools (MetaboAnalyst, IMPaLA, paintOmics) excel at enrichment statistics but provide limited capacity for exploratory network construction that preserves experimental context while leveraging pathway knowledge. PMconv addresses this gap by enabling users to bidirectionally map between experimentally detected proteins and metabolites through HMDB pathway annotations, visualize the resulting associations as integrated networks incorporating high-confidence protein–protein interactions from STRING, and interactively filter predictions by metabolite origin, molecular class, and literature support. We introduced PMconv as a complementary, mechanism-grounded baseline rather than a replacement for data-driven integrators.

## 2. Results

### 2.1. PMconv: An Interactive Platform for Proteometabolomic Network Analysis

To support pathway-based analysis of proteomic and metabolomic data, we developed PMconv, a web-based application that facilitates bidirectional conversion between protein and metabolite identifiers based on their shared involvement in metabolic pathways. This functionality enables annotation-based comparison of proteomic and metabolomic datasets and the expansion of experimentally derived molecular lists by identifying additional biologically relevant entities.

**Figure 1:**
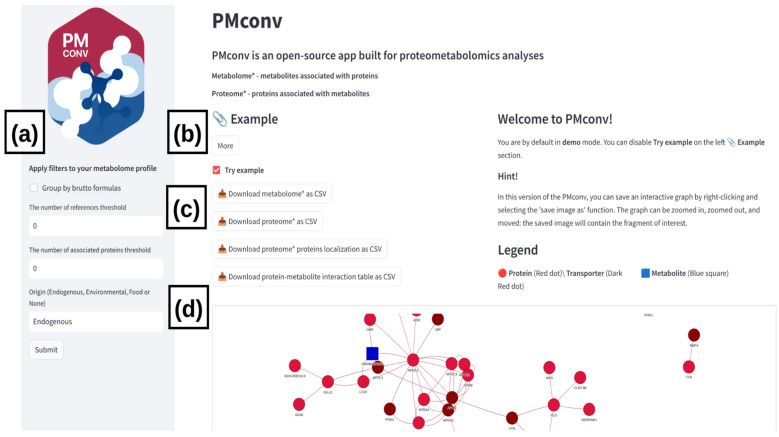
The PMconv user interface. (**a**) Panel for customizable filtering of metabolomic profiles, including options to group by molecular formula and specify data source or association thresholds. (**b**) A button to load example datasets for demonstration or testing purposes. (**c**) Download buttons for exporting analysis results in CSV files. (**d**) Interactive network visualization displaying proteins (red circles), transporters (dark red circles), and metabolites (blue squares), with zoom, pan, and node selection functionalities to explore proteometabolomic relationships.

The PMconv interface provides an interactive analytical environment tailored for the exploration of proteometabolomic relationships (see Figure 1). Key functionalities include customizable filtering of metabolomic profiles by molecular formula, compound class, and data origin (e.g., endogenous vs. environmental). Threshold parameters for inclusion of metabolite associations can be user-defined, thereby allowing refinement of the resulting molecular network to suit specific research contexts (see Figure 1a). PMconv currently supports UniProt (for proteins) and HMDB (for metabolites) identifiers, as it is primarily focused on the analysis of human-derived samples. However, the platform is under active development, and future versions are planned to expand its compatibility to support non-human organisms and alternative identifier systems. The user can use PMconv on both experimentally obtained lists or on one to obtain a list of related proteins or metabolites; however, the ability to construct an interaction network will be limited.

All protein–protein interactions in the network visualization are derived from the STRING database and filtered to include only experimentally validated physical interactions with confidence scores ≥ 0.9. Users can verify the specific confidence metrics for each interaction by referring to the STRING database using the protein identifiers shown in the network. This ensures the biological reliability of inferred protein networks. Importantly, experimentally detected metabolites are added to this high-confidence PPI network, enabling the visualization of combined protein–metabolite interaction networks.

The PMconv user interface (Figure 1) provides an intuitive and interactive analytical environment tailored for exploring proteometabolomic relationships. It includes a customizable filtering panel (Figure 1a) for metabolomic profiles, where users can group compounds by molecular formula and filter data by source (e.g., endogenous vs. environmental) or set thresholds for protein–metabolite associations. For convenience, users can load example datasets to explore the platform’s functionality (Figure 1b) or export results in .csv format for downstream analysis (Figure 1c). The exported table explicitly lists (Figure 1b) each metabolite of interest alongside its associated proteins, including enzymes, transporters and protein complexes, enabling straightforward inspection of proteometabolomic links. For tissue-specific analysis, we recommend using protein lists from curated databases like the Human Protein Atlas as input for the tool [16]. The main analytical output is an interactive network visualization (Figure 1d), where proteins are represented as red circular nodes, and metabolites as blue square nodes. Proteins annotated as transporters via the Transporter Classification Database (TCDB) [17] are rendered in a darker red to facilitate rapid identification of transport-mediated interactions. Users can explore the network through zoom, pan, and node selection functions, enabling detailed examination of molecular interactions, identification of key hubs, and characterization of metabolic pathway relationships.

### 2.2. Plasma Proteome and Metabolome Reveal Diverse Biological Functions

**Figure 2:**
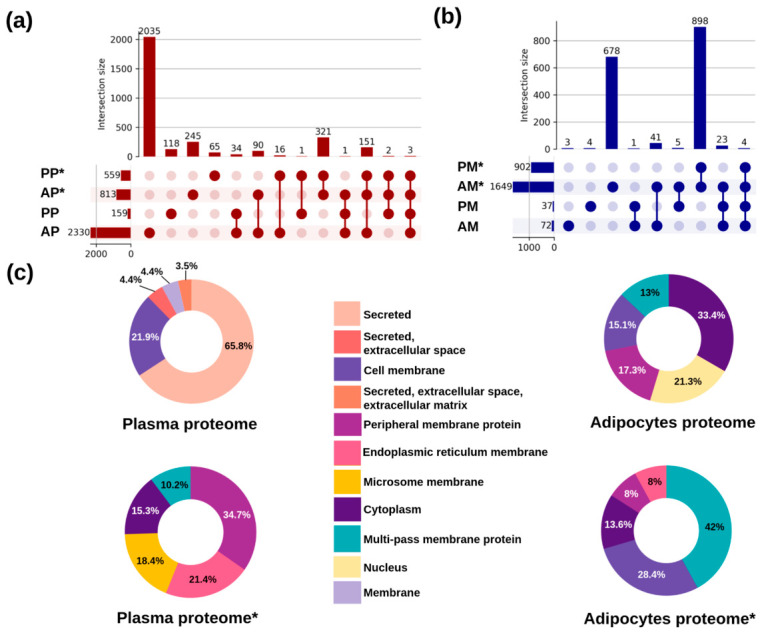
Mapping the metabolome to the proteome and the proteome to the metabolome of biomaterials. Comparison of lists of (**a**) metabolites and (**b**) proteins common to the experimentally obtained/associated data* (PP—plasma proteome, AP—adipocyte proteome, PP*—pathway-inferred plasma proteome*, AP*—pathway-inferred adipocyte proteome*) and experimentally obtained metabolome/pathway-inferred metabolome* (PM—plasma metabolome, AM—adipocyte metabolome, PM*—plasma metabolome*, AM*—adipocyte metabolome*). Intersection sets with zero objects were omitted. (**c**) Subcellular localization of unique experimentally identified proteins in blood plasma and adipocytes, as well as proteins of the proteome* of blood plasma and adipocytes.

To perform the integrative analysis of proteomic and metabolomic data, we applied PMconv to experimentally obtained protein lists in order to identify additional biologically relevant metabolites. Using 159 proteins identified in the blood plasma of obese individuals, PMconv retrieved 902 associated metabolites. Similarly, 352 proteins identified in healthy plasma samples yielded 995 metabolites, while 2330 proteins detected in adipocytes were associated with 1649 metabolites (see Figure 2a,b; Supplementary Table S1). These results demonstrate a clear trend: as the number of identified proteins increases, so does the number of associated metabolites. This pattern demonstrates PMconv’s ability to enumerate pathway-associated molecular candidates based on HMDB annotations, though the biological relevance of these candidates in specific tissue contexts requires experimental validation.

Conversely, PMconv was applied to experimentally detected metabolites to infer additional proteins. Specifically, 73 plasma metabolites yielded 65 unique associated proteins, and 134 adipocyte metabolites produced 245 proteins not initially observed in the experimental dataset. Interestingly, in contrast to the proteome-based expansion, the number of inferred proteins did not scale linearly with the number of metabolites. For instance, 33 metabolites were associated with 1055 proteins, whereas 37 metabolites yielded only 559 proteins (see Supplementary Table S1). This variation is likely influenced by the analytical platforms used for metabolomic profiling, as GC-MS and LC-MS/MS detect different classes of compounds, affecting metabolite representation and pathway mapping coverage [18,19]. Furthermore, the computational expansion offered by PMconv can partially overcome limitations inherent to specific experimental metabolomic platforms. For instance, the GC-MS approach used here is less suited for detecting large, polar, or thermally labile compounds such as certain nucleotides, peptides, or lipids. The metabolites inferred in the PM and AM sets may include such classes, as they are predicted based on protein associations rather than direct measurement. This provides a complementary layer of information, suggesting biochemical pathways and metabolites that might be missed by a targeted analytical method, thereby adding value to the pathway-based analysis.

To ensure biological consistency and minimize potential bias, we based our comparative analyses on molecular data from obese individuals for both plasma and adipocytes. This approach eliminates confounding effects arising from differences in health status and enables the identification of obesity-specific metabolic features. The alignment of proteomic and metabolomic profiles from matched samples provides a coherent framework for detecting disease-relevant molecular interactions, enriching our understanding of obesity-associated dysregulation and supporting the discovery of potential biomarkers and therapeutic targets.

To evaluate the performance of our pathway-based mapping strategy, we analyzed overlaps between experimentally obtained and pathway-inferred proteomic and metabolomic datasets derived from human plasma and adipose tissue. These data are summarized in Figure 2a,b. The size of the intersection is plotted on the Y-axis, and sets and their intersections are plotted on the X-axis. Figure 2a illustrates the intersection sizes among four proteomic datasets: experimentally derived plasma proteome (PP), adipocyte proteome (AP), and their pathway-inferred counterparts, plasma proteome* (PP*) and adipocyte proteome* (AP*). The largest individual dataset was the AP group, containing 2330 unique proteins. Notably, 2035 of these were found exclusively in AP, highlighting a significant number of unique identifiers in adipocytes. In contrast, the PP dataset included 159 unique proteins, while PP* and AP* contained 559 and 813 proteins, respectively. The most prominent intersection involved 321 common proteins shared between AP and AP*, demonstrating a relatively high overlap within the adipocyte-specific datasets. However, the intersection between PP and PP* included only 1 shared protein (0.8% overlap), indicating substantial divergence. This limited overlap reflects the context-independent nature of HMDB pathway annotations, which aggregate protein–metabolite associations across diverse tissues, species, and physiological conditions, whereas experimental measurements capture the specific molecular composition of the studied biological sample.

Figure 2b compares intersections among four metabolomics datasets: the experimentally detected plasma metabolome (PM) and adipocyte metabolome (AM), along with their pathway-based projections (PM* and AM*). The analysis reveals minimal direct overlap between experimental and computationally derived datasets—there is no overlap between PM and PM*, while the proteome comparison shows similarly low convergence (0.8% between PP and PP*). The observed minimal overlap between the directly measured plasma metabolome (PM) and the pathway-inferred plasma metabolome (PM*) likely reflects the fundamental biological distinction between intracellular and extracellular compartments. While PM represents metabolites exported into the plasma from cellular metabolism, PM* reflects metabolites associated with proteins present in the plasma, which may participate in ongoing membrane and extracellular processes. This is consistent with the larger overlaps observed for the cellular samples (AP/AP* and AM/AM*), where both measurement and inference primarily target the same intracellular compartment.

For both blood plasma and adipocytes, differences are observed between proteomes and proteomes*—sets of proteins associated with metabolites. Figure 2c presents the subcellular distribution of proteins unique to plasma and adipocyte proteomes, as well as those inferred via pathway mapping (proteome* datasets). Among experimentally detected plasma proteins, 65.8% were secreted, whereas the most common subcellular localizations in the plasma proteome* were peripheral membrane proteins (34.7%) and cell membrane proteins (21.4%). In adipocytes, the proteome was enriched in secreted and extracellular space proteins (33.4% and 21.3%, respectively), while the proteome* was dominated by multi-pass membrane proteins (42%) and cell membrane proteins (28.4%). According to these observations, the high abundance of common metabolites from plasma metabolome* and adipocyte metabolome* is most likely achieved through cell membrane proteins (see Figure 2c).

Importantly, these subcellular localization profiles were derived specifically for the subset of proteins associated with the shared metabolites between PM* and AM*. Thus, the observed high overlap of pathway-inferred metabolites is likely mediated by membrane-associated proteins present in both tissues, supporting the biological relevance of the shared metabolic features (see Figure 2c).

To assess the similarity between the experimental and pathway-inferred datasets, we calculated the Tanimoto index (T-index), a normalized metric for proportional overlap. Comparison of experimental and pathway-derived proteome profiles revealed complete differences between the experimental plasma and adipocyte proteomes (T-index << 0.01, Figure S2a), consistent with different biomaterial sources. Similarly, overlaps between each experimental proteome and its pathway-inferred counterpart were minimal (T-index << 0.01–0.08), indicating a substantial discrepancy between actual protein measurements and protein-derived metabolic associations.

Interestingly, the two pathway-inferred proteomes showed moderate similarity (T-index = 0.53, Figure S2a), suggesting some conserved metabolic associations across different tissues. In metabolomics, experimental plasma and adipocyte metabolomes showed moderate overlap (T-index = 0.35, Figure S2b), while PP* and AP* sets exhibited moderate overlap (T-index = 0.55, Figure S2b). Notably, overlaps between experimental and pathway-inferred datasets within the same tissue remained low (T-index << 0.01–0.04). The observed discrepancy between experimental and computational inferences highlights their fundamentally different biological scopes. Low Tanimoto overlaps between experimental and pathway-derived datasets are an expected outcome, given that reference databases are context-independent and catalog all theoretically documented interactions, while experimental data reflect only condition-specific molecular profiles. This divergence underscores the complementary value of both approaches rather than indicating a methodological shortcoming. Several concrete factors likely contribute to the limited overlap between measured and predicted metabolite sets. First, LC-MS-based metabolomics is inherently sensitive to pre-analytical variables, such as sample collection timing, storage conditions, and extraction protocols, which can lead to substantial metabolite degradation or selective loss of labile species [20]. Second, annotation bottlenecks persist: many detected features remain unidentified due to incomplete spectral libraries, isomeric ambiguity, or lack of authentic standards, meaning that biologically relevant metabolites may be present in the raw data but absent from the final reported lists [21]. Third, pathway databases themselves are incomplete and biased toward well-studied metabolic routes, so pathway-driven predictions may include metabolites that are biologically plausible yet undetected in a given experimental context due to coverage gaps. Together, these technical and knowledge-base limitations can systematically reduce the observable intersection between empirical and computationally inferred metabolomes, without necessarily reflecting false predictions. Thus, pathway-based annotation may identify both biologically relevant molecules below detection limits and pathway members not active in the specific tissue or condition studied.

### 2.3. PMconv Integrates STRING and HMDB for Biological Process Interpretation

**Figure 3:**
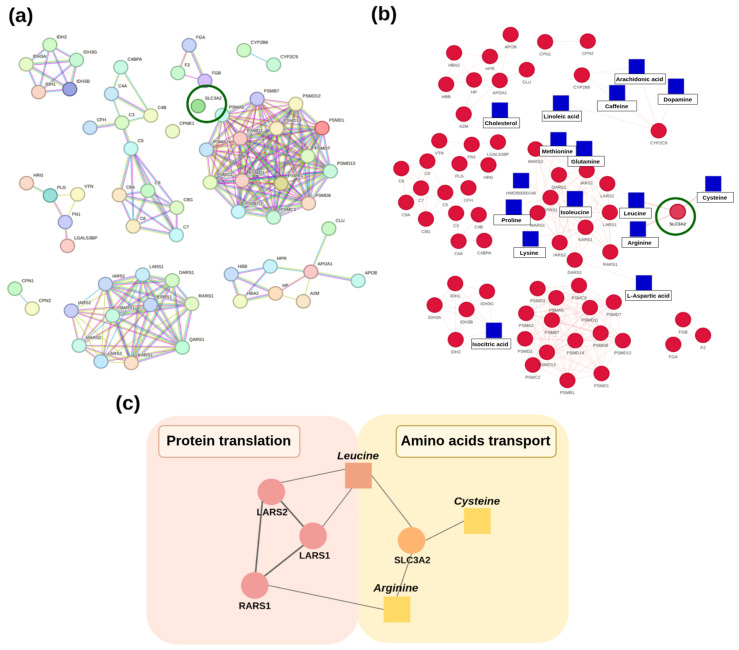
Networks of (**a**) protein–protein interactions obtained using STRING and (**b**) protein–metabolite pathway associations constructed using PMconv (red circles—proteins, blue squares—metabolites). The networks represent experimentally verified physical interactions (strict cutoffs of interactions according to the STRING database) of experimentally identified proteins in blood plasma (PP) and adipocytes (AP), with metabolites experimentally found in both studied biomaterials (PM and AM). Circled in green is the SLC3A2 protein, which was integrated into an interaction network through connections with two metabolites. (**c**) Protein–metabolite interactions of proteins LARS1, LARS2 and RARS1 and protein SLC3A2. Pink circles are blood plasma proteins, orange circles are adipocyte proteins, the salmon square is a metabolite experimentally found in both blood plasma and adipocytes, and yellow squares are metabolites experimentally found in adipocytes.

As was shown in the previous section, the PP* and AP* proteins have a different cellular localization from the PP and AP proteins. In addition to differences in cellular localization, pathway-derived proteins* perform different biological functions (see Table 1). Over-Representation Analysis (ORA) was conducted using WebGestalt to identify biological pathways significantly enriched in the protein sets. Statistically significant results (adjusted *p* < 0.05) are summarized in Table 1 for both plasma and adipocyte proteomes. PP proteins are characterized by processes associated with humoral immune cascades, such as reactions of the complement system (see Table 1a). PP* proteins mainly have connections with cellular processes of xenobiotic metabolism, amino acid transport and translation. This is consistent with the data in Figure 3c, which shows predominant localization in the peripheral cell membranes, microsomes and cytoplasm for these proteins.

For AP proteins, the processes of regulation of protein expression at the translation level, as well as the immune reaction of neutrophil degranulation, were mainly represented, as in PP* (see Table 1b). Compared to PP*, AP* proteins were associated with metabolic processes of energy metabolism, namely the biological oxidations, metabolism of amino acids and derivatives, metabolism of lipids and metabolism of carbohydrates. Processes associated with translation and activation of peroxisomes were less represented. The PP* and AP* sets exhibited moderate overlap (Tanimoto index = 0.55), driven primarily by proteins involved in translation-related processes (see Figure S2a and Table 1).

Previously, functional analysis of the experimentally determined plasma metabolome allowed Kiseleva O. and coauthors to reliably identify the processes of ammonia recycling, urea cycle, glycine and serine metabolism, glucose–alanine cycle and alanine metabolism [22]. Direct functional analysis of blood plasma metabolites does not allow us to determine the interrelated biological processes in which the proteins associated with these metabolites are involved. Using the converter, it was possible to determine cellular processes that can be limited by experimentally determined metabolites in the blood plasma.

To explore the biological relevance of shared features between experimentally and pathway-derived datasets, we examined the overlap in enriched pathways, particularly those identified in the pathway-derived protein sets. In the plasma proteome* (PP*), notable pathways included “tRNA aminoacylation” and “Metabolism of amino acids and derivatives”—both consistent with pathway annotations of the experimentally identified plasma metabolites, which were predominantly amino acids. The observed divergence between experimentally determined and pathway-derived proteomes primarily reflects fundamental biological constraints related to cellular compartmentalization and the context-independent nature of pathway databases. Biochemical knowledge bases aggregate enzyme–metabolite relationships across all tissues and subcellular compartments without specifying spatial localization. Consequently, the detection of a metabolite in a biological fluid such as plasma does not imply co-localization of all associated metabolic enzymes; rather, it typically indicates active membrane transport, systemic metabolic crosstalk, or secretion from distal tissues. The pathway-derived proteome* therefore represents a knowledge-inferred landscape of potential biochemical associations rather than a direct prediction of the local proteomic composition. While this limits the tool’s applicability for compartment-specific proteome reconstruction, it provides a structured framework for identifying systemic metabolic themes, potential upstream regulatory processes, and cross-tissue signaling networks that may be modulated by the observed metabolite profile. Accordingly, PMconv should be applied as an exploratory resource for mapping metabolite-driven biological contexts, with the explicit understanding that inferred connections reflect curated biochemical possibilities that require spatial filtering and orthogonal experimental validation to confirm compartment-specific relevance. To facilitate this spatial contextualization, PMconv output automatically includes subcellular and tissue compartment annotations for all mapped proteins, as curated by the Human Protein Atlas [16], enabling users to filter and prioritize interactions that align with the biological context of their experimental system (see Table S8).

The STRING web application, while widely used for protein–protein interaction (PPI) analysis, does not incorporate protein–metabolite interactions, which may limit the biological insight into metabolically relevant protein functions. To address this, PMconv was used to construct interaction networks using proteins and metabolites involved in the biological pathways listed in Table 1, focusing strictly on physical interactions experimentally validated in STRING (e.g., crystallography, immunoprecipitation), with the highest confidence level (0.9). Importantly, proteins and metabolites were included only if they were experimentally identified in either blood plasma or adipocytes, thereby preserving the biological integrity of specific biomaterial data.

The goal of this analysis was to explore potential cross-tissue interactions, particularly between secreted adipocyte proteins and plasma components, reflecting physiological crosstalk, for example, endocrine signaling or lipid metabolism. Figure 3a shows that the STRING PPI network forms nine distinct clusters, with two plasma proteins (CPNE1 and SLC3A2) remaining unclustered. However, when experimentally detected metabolites from both tissues were incorporated into the network (Figure 3b), the SLC3A2 protein became connected via leucine and arginine to the aminoacyl-tRNA synthetases LARS1, LARS2, and RARS1—proteins associated with the enriched “tRNA aminoacylation” pathway. Notably, leucine was found in both plasma and adipocytes, and arginine in adipocytes only (Figure 3c). Importantly, the detection of leucine in plasma does not require or imply the presence of tRNA aminoacylation pathway proteins in the bloodstream; rather, it reflects active membrane transport and systemic metabolite distribution that functionally bridge spatially separated metabolic modules. When such primarily intracellular enzymes are experimentally detected in circulation, this typically reflects physiological cellular turnover, extracellular vesicle-mediated release, or regulated secretion—processes that introduce intracellular proteins into plasma without conferring extracellular aminoacylation activity.

Previous work showed that leucine/isoleucine metabolites were directly related to body mass index and were generally associated with obesity [23]. PMconv identified a shared pathway annotation linking the major cellular leucine regulators LARS1 and SLC3A2. Among the proteins initially unconnected in the STRING PPI network, SLC3A2 was selected for detailed analysis because it is a known amino acid transporter with established roles in leucine uptake. Leucine was detected in both plasma and adipocyte metabolomes, and SLC3A2 showed no direct protein–protein interactions in STRING despite being experimentally detected, suggesting potential functional connections mediated through shared metabolites rather than direct protein binding. These proteins were shown to play a regulatory role in the pathogenesis of diabetes and obesity [24,25,26].

## 3. Discussion

The current state of molecular knowledge—spanning genes, proteins, and metabolites—presents a challenge akin to reconstructing a functional electronic device from a disorganized collection of microcircuits without a schematic. Isolated descriptions of individual components, while valuable, fail to capture the emergent properties of the integrated system. Much like attempting to deduce the function of a radio solely by cataloging its disparate parts, this reductionist approach overlooks the critical interactions and higher-order logic that govern biological function.

Similarly, in the 21st century, researchers are faced with a multitude of objects: thousands of genes, millions of proteoforms, hundreds of metabolites, and many interactions between these objects. Such diversity does not make it possible to move from describing individual objects to describing the mechanisms of functioning of a living cell.

One possible strategy for understanding system-level behavior is to identify and analyze relationships among observable entities. In this context, the development of tools that connect different molecular types is essential for advancing our understanding.

It has been repeatedly noted that despite the very high sensitivity and development of analytical methods, proteomics has brought very few biomarkers to practical medicine [27]. Not being able to multiply low-molecular compounds and proteins, in our work, we showed that molecular objects can be amplified not experimentally, but in the information space (in silico). Of course, the expansion of the space of protein and low-molecular objects is accompanied by a loss of compartment-specific specificity, as the mapping reflects curated biochemical knowledge rather than direct spatial co-localization. For instance, the detection of amino acids in plasma does not imply that their corresponding metabolic enzymes are present in the bloodstream; rather, it typically signals active membrane transport, systemic metabolic crosstalk, or tissue-specific secretion. Consequently, the pathway-derived proteome* should not be interpreted as a predictor of local proteomic composition, but as a framework for reconstructing systemic metabolic themes and upstream regulatory cascades. This perspective shifts the search for biomarkers from single molecules or co-localized sets toward functionally connected molecular networks operating across biological compartments.

The problem of analyzing the results of such transformations is associated with the increase in objects in the search field—their number increases, and the found relationships are presented in the form of a difficult-to-interpret ball (“hairball”). To analyze relationships, it is necessary to integrate visualization tools in the form of networks. At the time of constructing relationships, the noise level is reduced—single relationships are removed from consideration, and only confirmed and repeatable events are visualized, thereby reducing the complexity of the visual representation. An example of a visualization system is STRING, proposed more than 10 years ago [28].

The PMconv app extends the STRING network by incorporating metabolite data. While STRING constructs protein–protein interaction networks, PMconv enhances these networks by associating a list of metabolites with each protein node. These protein–metabolite relationships are determined using HMDB data and validated against experimental datasets of detected proteins and metabolites. This approach allows isolated clusters of interacting proteins to be interconnected into a unified network through shared metabolite associations. This integration addresses a practical challenge in multi-omics analysis: while proteins without direct physical interactions may appear functionally unrelated in PPI networks, their shared association with specific metabolites can reveal functional relationships operating through metabolic flux rather than protein binding. The SLC3A2-LARS1 example (Figure 3) illustrates this principle: both proteins participate in leucine homeostasis (transport and tRNA charging, respectively) but do not physically interact, making their functional connection invisible to PPI-only analysis.

The network of protein–metabolite relationships constructed in this way differs significantly both in objects and in relationships from the networks built by other tools. We compared our resulting network of protein interactions (STRING, adipocytes) and protein–metabolite interactions (PMconv) and visualized relationships for proteins and adipocyte metabolites from the MetalinksDB resource. This resource focuses on relationships of interest in the context of drug metabolism and pharmacology [15]. The objects of such a network are studied and characterized protein targets. Networks built using PMconv are likely to be a source of new targets. It is important to note that this kind of representation of an information array about proteins and metabolites coincides with the representation of biochemical processes in KEGG and is inherited by the HMDB data structure. Let us emphasize once again that in our algorithm, there is no pattern recognition, no other elements of machine learning, much less neural networks that could somehow learn or adjust to the way data is presented. These are exclusively informational sections based on the results of the experiment, mapped to the current level of knowledge about the interaction of proteins and metabolites.

Recent advances in machine learning have substantially expanded the toolkit for multi-omics integration, with graph neural networks, transformers, and large language models enabling sophisticated inference of molecular interactions—from circRNA–miRNA regulatory networks in cancer [29,30] to target prioritization in drug discovery [31] and spatially resolved cell–cell communication mapping via approaches such as SpaCCC [32]. While these methods achieve high predictive performance, their application to primary proteome–metabolome integration faces persistent challenges: limited interpretability of latent graph or attention representations, sensitivity to modality-specific data structures, and dependence on large, well-annotated training sets. PMconv is designed to complement this ecosystem by offering a mechanism-grounded, pathway-centric workflow that prioritizes explicit mapping traceability and biological interpretability, thereby supporting hypothesis generation and feature engineering for subsequent data-driven modeling.

In summary, PMconv enabled pathway-based annotation of proteomic and metabolomic datasets, enumerating pathway-associated candidates and suggesting potential interactions—including active membrane transport, metabolite trafficking, and systemic crosstalk—not readily captured through experimental data alone. Application of PMconv to human plasma and adipocyte datasets demonstrated its capacity to uncover shared molecular features, enhance network connectivity, and identify functionally relevant proteins and metabolites. PMconv enabled comparative pathway-based annotation of molecular profiles of different tissues and a shift in focus to systems analysis of the proteome and metabolome. We show that our tool PMconv coupled with molecular networking functionality can visualize the results of proteomics and metabolomics experiments. The integral form of representation of the proteome and metabolome is more consistent with the principles of cell organization. The next step is to identify significant changes in these kinds of networks with various pronounced pathological conditions or individual characteristics of the course of the disease. Observation of networks in a longitudinal format or when comparing cohorts serves as a new slice of molecular data in the aspect of personalized medicine for the functional analysis of molecular profiles and the generation of hypotheses about the mechanisms of disease formation.

## 4. Materials and Methods

### 4.1. Initial Data

Lists of proteins associated with obesity obtained by LC-MS/MS and metabolites detected by GC-MS and GCxGC-MS were selected. These methods were used to obtain molecular profiles of human blood plasma samples and adipocytes from various localizations (visceral adipose tissue, subcutaneous abdominal adipose tissue and gluteofemoral adipose tissue). The multiple identifiers of detected metabolites in the three types of adipocytes were merged. Lists of proteins and metabolites obtained by LC-MS/MS from human blood plasma of obese patients were used to test the performance of the developed proteometabolomic tool. The data used in this work were taken from the publications of Kiseleva O.I. et al. [22,23] and Raajendiran A. et al. [33]. The lists of proteins and metabolites used for the analysis are presented in Supplementary Tables S1–S7. All these lists of metabolites and proteins are experimentally obtained proteomic and metabolomic datasets.

### 4.2. Estimation of the Degree of Similarity of Sets

The Tanimoto index [34] was used to assess the degree of similarity between two sets. The Tanimoto index was calculated according to formula (1):

K = C/(A + B − C),(1)

where K is the Tanimoto index (values from 0 to 1; the closer to 1, the greater the similarity of the sets), A and B are the numbers of protein or metabolite identifiers in the two sets, and C is the number of shared protein or metabolite identifiers in the two sets.

### 4.3. Proteometabolomic Converter PMconv

PMconv (Proteome–Metabolome converter) is a web-based application (https://pmconv.streamlit.app/, accessed on 1 June 2026) for converting protein identifiers into identifiers of metabolites associated with these proteins and vice versa. The converter was based on associations between proteins and metabolites established in the HMDB. The application was developed in Python (3.10) using the Streamlit framework.

**Figure 4:**
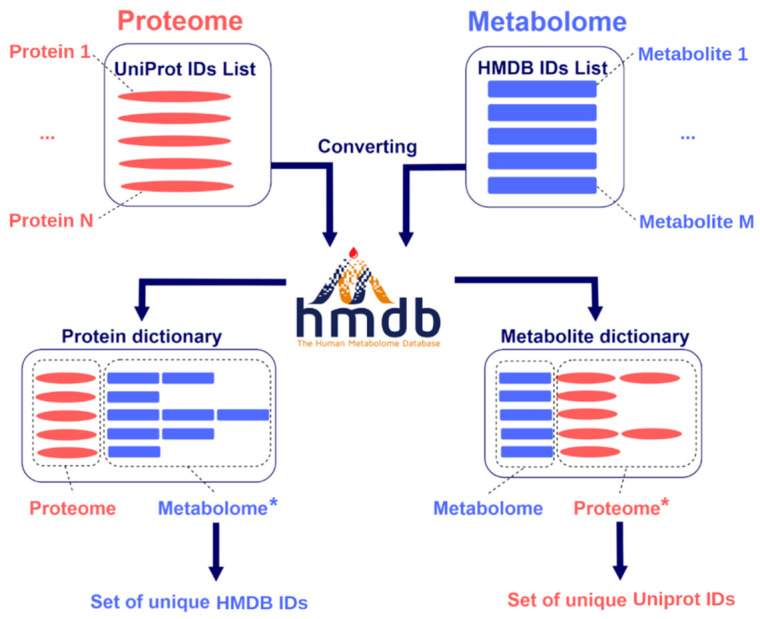
PMconv workflow: converting protein and metabolite identifiers using the HMDB. Red elements—proteins (UniProt IDs or Proteome), blue elements—metabolites (HMDB IDs or Metabolome). Metabolome*/Proteome*—list of unique identifiers of metabolites/proteins involved in the same metabolic pathways as the query identifiers.

The workflow of PMconv is shown in Figure 4. The converter receives a list of protein identifiers (UniProt ID, see Figure 4) as input data. Alternatively, the query can be a list of metabolite identifiers (HMDB ID, see Figure 4), which can be used to search for associated objects in the metabolite database. As an intermediate result of PMconv, when using the list of metabolites, we obtain a dictionary in which each metabolite identifier is associated with a set of protein identifiers of the same metabolic pathway. Similarly, when using a list of proteins as input, a protein dictionary can be created where each protein identifier has a corresponding list of associated metabolite identifiers (see Figure 4).

The output of PMconv is a list of unique identifiers of metabolites (“Metabolome*”) involved in the same metabolic pathways as the proteins from the query. Similarly, the set (“Proteome*”) consists of proteins associated, according to HMDB, with the list of metabolites specified as a query to PMconv. Although modern mass spectrometry approaches—such as MS/MS fragmentation and chromatographic separation—enable high-confidence identification of metabolites and discrimination between isomers, in practical applications (particularly when working with public datasets or low-resolution platforms), identifications may still be limited to molecular formulas [35]. To accommodate such cases and improve interpretability, PMconv offers a filtering option that aggregates metabolites with identical brutto-formulas into a single node, regardless of specific HMDB identifiers. This strategy is particularly valuable when analyzing data with limited identification depth (e.g., based only on accurate mass), when aiming to highlight shared chemical or functional groups involved in metabolic processes, or when simplifying the visualization and interpretation of large molecular networks in which isomeric compounds often participate in the same pathways. The use of brutto-formulas to represent metabolites, as shown in Figure S1, leads to a significant reduction in the dimensionality of the protein–metabolite network. In this work, for all sets of metabolites (plasma and adipocytes metabolome and metabolome*), the following filters were applied: endogenous origin, the number of associated proteins > 1, the number of articles > 1. All metabolites were merged by brutto-formulas.

The PMconv functionality allows the comparison of the received lists of identifiers to process the results of postgenomic (proteomic and metabolomic) profiling of samples. PMconv capabilities allow visualization of the results obtained in the form of a network of relationships and comparison of the results obtained with STRING data [36] for functional interpretation of data. Network visualization parameters: Protein–protein interactions were retrieved from the STRING database (v12.0) with a confidence score ≥ 0.9, limited to experimentally validated physical interactions (excluding text-mining, co-expression, and predicted associations). This stringent filtering ensures that visualized connections represent experimentally confirmed direct binding or complex formation. The PMconv results can be easily combined with downstream statistical analysis or machine learning models. To account for spatial context, we integrated subcellular localization annotations from the Human Protein Atlas [16], assigning each protein its major compartment(s) based on immunofluorescence and RNA-seq evidence. This compartment metadata enables optional spatial filtering of the interaction network, allowing users to prioritize connections consistent with the biological context of their experimental sample. Additionally, transporter status for each protein was annotated using the Transporter Classification Database (TCDB) [17]. Only entries with experimentally supported or computationally predicted TCDB classifications were retained to ensure high-confidence functional labeling during network visualization and downstream filtering.

### 4.4. Over-Representation Analysis via WebGestalt

We performed over-representation analysis (ORA) using WebGestalt (WEB-based GEne SeT AnaLysis Toolkit) to identify significantly enriched biological pathways and functional categories among our identified and pathway-inferred proteins [37]. For this analysis, we used the Reactome reference database [38].

The analysis parameters were set as follows: we considered results statistically significant if they had an adjusted *p*-value < 0.05 (using Benjamini–Hochberg FDR correction), required a minimum of 5 entities per category, and used appropriate reference backgrounds (the human genome).

### 4.5. Limitations and Interpretive Considerations

Limitations and interpretive considerations: PMconv performs database lookup in HMDB without tissue-specific filtering, subcellular localization constraints, or probabilistic confidence scoring. Pathway co-membership indicates potential functional relationships but does not predict molecular co-occurrence in specific biological samples. Pathway-inferred proteins and metabolites should be interpreted as hypothesis-generating candidate lists requiring experimental validation or integration with tissue-specific expression data (e.g., GTEx, Human Protein Atlas [16]) rather than predictions of molecular presence.

## Figures and Tables

**Table 1:** Top 5 Reactome biological pathways associated with proteins.

**(a)**					
**Plasma proteome**	**N.P. ^1^**	***p*** **-value**	Plasma proteome*^,2^	**N.P.**	***p*** **-value**
Regulation of Complement cascade	18	1.2 × 10^−27^	Metabolism of amino acids and derivatives	109	4.8 × 10^−60^
Complement cascade	19	1.4 × 10^−27^	tRNA Aminoacylation	30	1.2 × 10^−31^
Platelet degranulation	22	5.8 × 10^−25^	Phase I—Functionalization of compounds	39	6.9 × 10^−26^
Response to elevated platelet cytosolic Ca^2+^	22	1.4 × 10^−24^	Xenobiotics	20	3.5 × 10^−23^
Hemostasis	33	4.4 × 10^−21^	Cytochrome P450—arranged by substrate type	29	3.9 × 10^−22^
**(b)**					
**Adipocytes proteome**	**N.P. ^1^**	***p*** **-value**	Adipocytes proteome*^,2^	**N.P.**	***p*** **-value**
Metabolism of amino acids and derivatives	196	1.5 × 10^−57^	Metabolism of amino acids and derivatives	137	3.9 × 10^−69^
Neutrophil degranulation	215	7.6 × 10^−49^	Metabolism of lipids	145	1.3 × 10^−35^
Translation	155	9.0 × 10^−47^	Biological oxidations	67	6.9 × 10^−28^
Regulation of expression of SLITs and ROBOs	111	5.6 × 10^−45^	tRNA Aminoacylation	29	2.3 × 10^−25^
The citric acid (TCA) cycle and respiratory electron transport	113	1.0 × 10^−44^	Metabolism of carbohydrates	72	2.7 × 10^−23^

^1^ N.P.—The number of proteins associated with Reactome pathway according to UniProt DB (06_2024). ^2^ Plasma/Adipocytes proteome* (PP*/AP*) reflects knowledge-derived associations aggregated across biological contexts, whereas Plasma proteome/Adipocytes proteome (PP/AP) represents experimentally detected proteins in the specified compartment.

## Data Availability

The PMconv is available at https://pmconv.streamlit.app/. The source code was deposited in the GitHub repository https://github.com/Ministreliya131/PMconv (accessed on 1 June 2026).

## Supplementary Materials

The following supporting information can be downloaded at: https://www.mdpi.com/article/10.3390/ijms27115086/s1.

## References

[1] Ma’ayan A. (2011). Introduction to Network Analysis in Systems Biology. Sci. Signal..

[2] (2010). The Human Proteome Organization. A Gene-Centric Human Proteome Project. Mol. Cell. Proteom..

[3] Wishart D.S., Guo A., Oler E., Wang F., Anjum A., Peters H., Dizon R., Sayeeda Z., Tian S., Lee B.L. (2022). HMDB 5.0: The Human Metabolome Database for 2022. Nucleic Acids Res..

[4] Kanehisa M. (2000). KEGG: Kyoto Encyclopedia of Genes and Genomes. Nucleic Acids Res..

[5] Kamburov A., Cavill R., Ebbels T.M.D., Herwig R., Keun H.C. (2011). Integrated Pathway-Level Analysis of Transcriptomics and Metabolomics Data with IMPaLA. Bioinformatics.

[6] Karnovsky A., Weymouth T., Hull T., Tarcea V.G., Scardoni G., Laudanna C., Sartor M.A., Stringer K.A., Jagadish H.V., Burant C. (2012). Metscape 2 Bioinformatics Tool for the Analysis and Visualization of Metabolomics and Gene Expression Data. Bioinformatics.

[7] Liu T., Salguero P., Petek M., Martinez-Mira C., Balzano-Nogueira L., Ramšak Ž., McIntyre L., Gruden K., Tarazona S., Conesa A. (2022). PaintOmics 4: New Tools for the Integrative Analysis of Multi-Omics Datasets Supported by Multiple Pathway Databases. Nucleic Acids Res..

[8] Pang Z., Chong J., Zhou G., de Lima Morais D.A., Chang L., Barrette M., Gauthier C., Jacques P.-É., Li S., Xia J. (2021). MetaboAnalyst 5.0: Narrowing the Gap between Raw Spectra and Functional Insights. Nucleic Acids Res..

[9] Huang H., Van Dullemen L.F.A., Akhtar M.Z., Faro M.-L.L., Yu Z., Valli A., Dona A., Thézénas M.-L., Charles P.D., Fischer R. (2018). Proteo-Metabolomics Reveals Compensation between Ischemic and Non-Injured Contralateral Kidneys after Reperfusion. Sci. Rep..

[10] Halter D., Goulhen-Chollet F., Gallien S., Casiot C., Hamelin J., Gilard F., Heintz D., Schaeffer C., Carapito C., Van Dorsselaer A. (2012). In Situ Proteo-Metabolomics Reveals Metabolite Secretion by the Acid Mine Drainage Bio-Indicator, Euglena Mutabilis. ISME J..

[11] Mujahid M., Prasuna M.L., Sasikala C., Ramana C.V. (2015). Integrated Metabolomic and Proteomic Analysis Reveals Systemic Responses of *Rubrivivax Benzoatilyticus* JA2 to Aniline Stress. J. Proteome Res..

[12] Sadhu S., Rizvi Z.A., Pandey R.P., Dalal R., Rathore D.K., Kumar B., Pandey M., Kumar Y., Goel R., Maiti T.K. (2021). Gefitinib Results in Robust Host-Directed Immunity Against Salmonella Infection Through Proteo-Metabolomic Reprogramming. Front. Immunol..

[13] Shekhar S., Agrawal L., Mishra D., Buragohain A.K., Unnikrishnan M., Mohan C., Chakraborty S., Chakraborty N. (2016). Ectopic Expression of Amaranth Seed Storage Albumin Modulates Photoassimilate Transport and Nutrient Acquisition in Sweetpotato. Sci. Rep..

[14] Bhattacharya S.K. (2019). Methods in molecular biology. Metabolomics: Methods and Protocols.

[15] Farr E., Dimitrov D., Schmidt C., Turei D., Lobentanzer S., Dugourd A., Saez-Rodriguez J. (2024). MetalinksDB: A Flexible and Contextualizable Resource of Metabolite-Protein Interactions. Brief. Bioinform..

[16] Uhlén M., Fagerberg L., Hallström B.M., Lindskog C., Oksvold P., Mardinoglu A., Sivertsson Å., Kampf C., Sjöstedt E., Asplund A. (2015). Tissue-Based Map of the Human Proteome. Science.

[17] Saier M.H. (2006). TCDB: The Transporter Classification Database for Membrane Transport Protein Analyses and Information. Nucleic Acids Res..

[18] Kiseleva O., Kurbatov I., Ilgisonis E., Poverennaya E. (2021). Defining Blood Plasma and Serum Metabolome by GC-MS. Metabolites.

[19] Mofokeng N.N., Madikizela L.M., Tiggelman I., Chimuka L. (2024). Chemical Profiling of Paper Recycling Grades Using GC-MS and LC-MS: An Exploration of Contaminants and Their Possible Sources. Waste Manag..

[20] Johnson C.H., Gonzalez F.J. (2012). Challenges and Opportunities of Metabolomics. J. Cell. Physiol..

[21] Nguyen Q.-H., Nguyen H., Oh E.C., Nguyen T. (2024). Current Approaches and Outstanding Challenges of Functional Annotation of Metabolites: A Comprehensive Review. Brief. Bioinform..

[22] Kiseleva O.I., Pyatnitskiy M.A., Arzumanian V.A., Kurbatov I.Y., Ilinsky V.V., Ilgisonis E.V., Plotnikova O.A., Sharafetdinov K.K., Tutelyan V.A., Nikityuk D.B. (2024). Multiomics Picture of Obesity in Young Adults. Biology.

[23] Kiseleva O.I., Arzumanian V.A., Poverennaya E.V., Pyatnitskiy M.A., Ilgisonis E.V., Zgoda V.G., Plotnikova O.A., Sharafetdinov K.K., Lisitsa A.V., Tutelyan V.A. (2021). Does Proteomic Mirror Reflect Clinical Characteristics of Obesity?. J. Pers. Med..

[24] Raevsky A., Kovalenko O., Bulgakov E., Sharifi M., Volochnyuk D., Tukalo M. (2024). Developing a Comprehensive Solution Aimed to Disrupt LARS1/RagD Protein-Protein Interaction. J. Biomol. Struct. Dyn..

[25] Kahlhofer J., Teis D. (2023). The Human LAT1–4F2hc (SLC7A5–SLC3A2) Transporter Complex: Physiological and Pathophysiological Implications. Basic Clin. Pharmacol. Toxicol..

[26] Eom J., Choi J., Suh S.-S., Seo J.B. (2022). SLC3A2 and SLC7A2 Mediate the Exogenous Putrescine-Induced Adipocyte Differentiation. Mol. Cells.

[27] Ye X., Blonder J., Veenstra T.D. (2009). Targeted Proteomics for Validation of Biomarkers in Clinical Samples. Brief. Funct. Genom. Proteomic..

[28] Snel B., Lehmann G., Bork P., Huynen M.A. (2000). STRING: A Web-Server to Retrieve and Display the Repeatedly Occurring Neighbourhood of a Gene. Nucleic Acids Res..

[29] Wei M.-M., Wang L., Zhao B.-W., Su X.-R., You Z.-H., Huang D.-S. (2025). Integrating Transformer and Graph Attention Network for circRNA-miRNA Interaction Prediction. IEEE J. Biomed. Health Inform..

[30] Wei M., Wang L., Su X., Zhao B., You Z. (2026). Multi-Hop Graph Structural Modeling for Cancer-Related circRNA-miRNA Interaction Prediction. Pattern Recognit..

[31] Wang X., Wang C., Ji B., Wang J., Zheng M., Song L., Peng S., Shang X. (2026). Multimodal Pre-Training Models of Molecular Representation for Drug Discovery. Natl. Sci. Rev..

[32] Ji B., Wang X., Qiao D., Xu L., Peng S. (2024). SpaCCC: Large Language Model-Based Cell-Cell Communication Inference for Spatially Resolved Transcriptomic Data. Big Data Min. Anal..

[33] Raajendiran A., Krisp C., Souza D.P.D., Ooi G., Burton P.R., Taylor R.A., Molloy M.P., Watt M.J. (2021). Proteome Analysis of Human Adipocytes Identifies Depot-Specific Heterogeneity at Metabolic Control Points. Am. J. Physiol.-Endocrinol. Metab..

[34] Rogers D.J., Tanimoto T.T. (1960). A Computer Program for Classifying Plants: The Computer Is Programmed to Simulate the Taxonomic Process of Comparing Each Case with Every Other Case. Science.

[35] Novoa-del-Toro E.M., Witting M. (2024). Navigating Common Pitfalls in Metabolite Identification and Metabolomics Bioinformatics. Metabolomics.

[36] Szklarczyk D., Kirsch R., Koutrouli M., Nastou K., Mehryary F., Hachilif R., Gable A.L., Fang T., Doncheva N.T., Pyysalo S. (2023). The STRING Database in 2023: Protein–Protein Association Networks and Functional Enrichment Analyses for Any Sequenced Genome of Interest. Nucleic Acids Res..

[37] Elizarraras J.M., Liao Y., Shi Z., Zhu Q., Pico A.R., Zhang B. (2024). WebGestalt 2024: Faster Gene Set Analysis and New Support for Metabolomics and Multi-Omics. Nucleic Acids Res..

[38] Milacic M., Beavers D., Conley P., Gong C., Gillespie M., Griss J., Haw R., Jassal B., Matthews L., May B. (2024). The Reactome Pathway Knowledgebase 2024. Nucleic Acids Res..

