# Supplementary material for: PMconv: How to Compare Proteomes and Metabolomes?
Source: Int J Mol Sci. 2026 Jun 4;27(11):5086. doi: 10.3390/ijms27115086 (PMC13256924; doi:10.3390/ijms27115086)
Supplement: Supplementary file 1 [file ijms-27-05086-s001.zip › PMconv_Supplementary_Materials.pdf]

Supplementary Materials

PMconv: how to compare proteomes and metabolomes?

Anna Kozlova[1,2,*], Elena Ponomarenko[1], Ekaterina Ilgisonis[1,2], Victor Tutelyan[3], Andrey Lisitsa[1,2]

*Corresponding author: Anna Kozlova

Email Address of the Corresponding Author: ministreliya13113@gmail.com

[1] Institute of Biomedical Chemistry, Moscow, 119121 Moscow, Russia

[2] Tyumen State University, 625003 Tyumen, Russia

[3] Federal Research Centre of Nutrition, Biotechnology and Food Safety, Russian Academy of Sciences, Moscow 109240, Russia

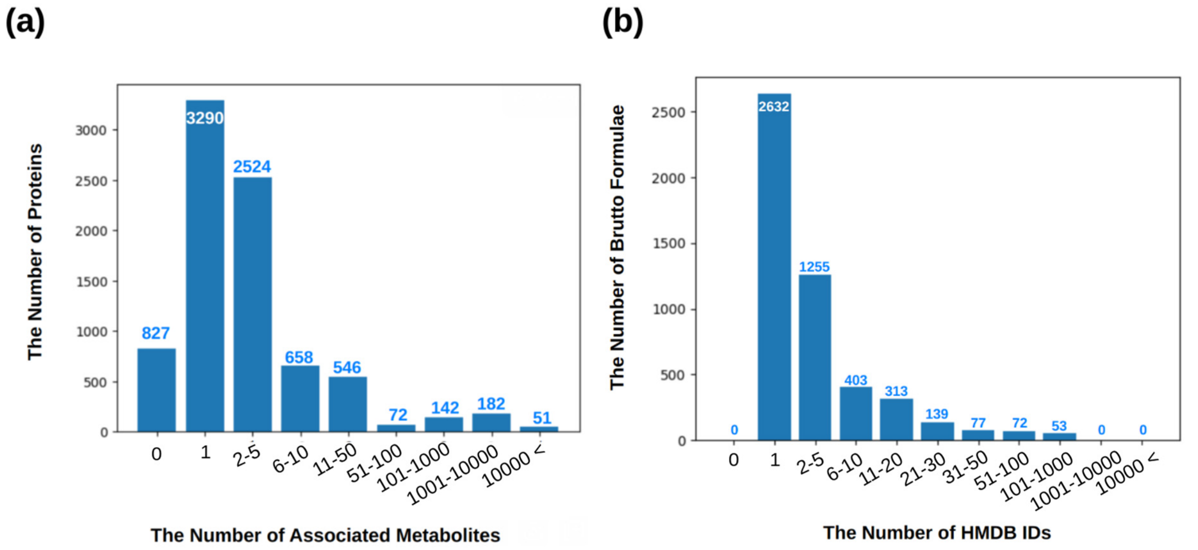

Figure S1. Histogram of associations according to HMDB data **(a)** between proteins and metabolites **(b)** and metabolites with the same brutto-formula.

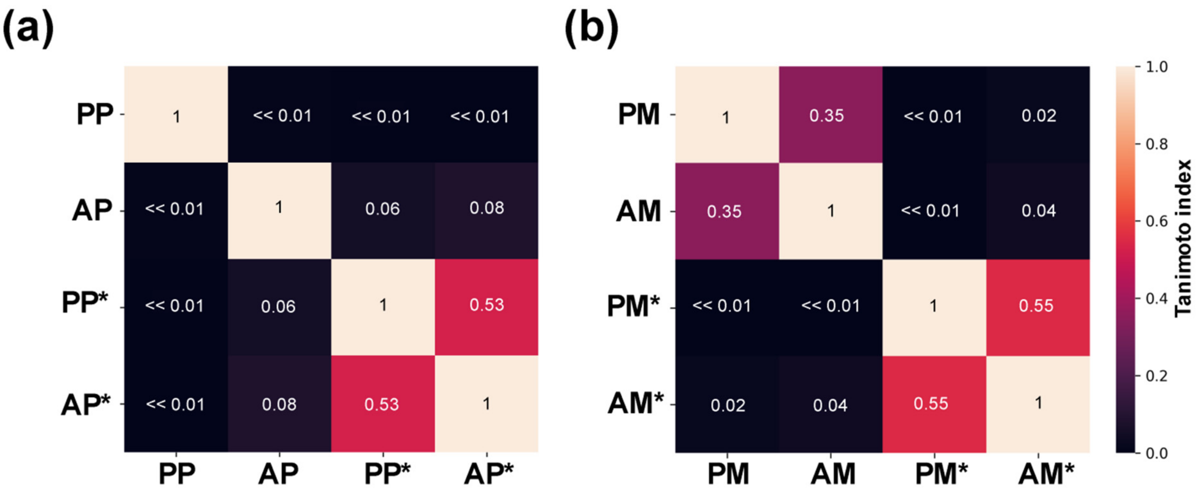

Figure S2. Heatmap of similarity between sets of **(a)** proteomes/proteomes* and **(b)** metabolomes/metabolomes*. **PM** – plasma metabolome, **PM*** – plasma metabolome*, **AM** – adipocytes metabolome, **AM*** – adipocytes metabolome*, **PP** – plasma proteome, **PP*** – plasma proteome*, **AP** – adipocytes proteome, **AP*** – adipocytes proteome*.
